# Translatability scoring in drug development: eight case studies

**DOI:** 10.1186/1479-5876-10-39

**Published:** 2012-03-07

**Authors:** Alexandra Wendler, Martin Wehling

**Affiliations:** 1 Institute of Experimental and Clinical Pharmacology and Toxicology Clinical Pharmacology Mannheim, Faculty of Medicine Mannheim, Ruprecht-Karls-University of Heidelberg, Maybachstr.14, D-68169 Mannheim, Germany

**Keywords:** Dabigatran, Ipilimumab, Gefitinib, Vilazodone, Latrepirdine, Semagacestat, Translatability Score, Translational medicine, Drug development

## Abstract

Translational medicine describes the transfer of basic *in vitro *and *in vivo *data into human applications. In the light of low rates of market approvals for new medical entities, better strategies to predict the risk of drug development should be used to increase output and reduce costs. Recently, a scoring system to assess the translatability of early drug projects has been proposed. Here eight drugs from different therapeutic areas have been subjected to a retrospective test-run in this system fictively located at the phase II-III transition. The scores gained here underline the importance of biomarker quality which is pivotal to decrease the risk of the project in all cases. This is particularly evident for gefitinib. The EGFR mutation status is a breakthrough biomarker to predict therapeutic success which made this compound clinically acceptable, and this is plausibly reflected by a considerable increase of the translatability score. For psychiatric and Alzheimer's drugs, and for a CETP-inhibitor, the lack of suitable biomarkers and animal models is reflected by a low translatability score, well correlating with the excessive translational risk in these areas. These case studies document the apparent utility of the scoring system, at least under retrospective conditions, as the scores correlate with the outcomes at the level of market approval. Prospective validation is still missing, but these case studies are encouraging.

## Introduction

Translational medicine is an important component of drug development and describes the conditions and prerequisites for the transfer of *in vitro *and *in vivo *findings into human applications [1], and should ultimately facilitate the development of new drugs. It is hoped that the "empty pipeline syndrome" (lack of innovation in drug industry, exceptions granted, e.g. oncology) could be treated by this (and other) means and the sequelae of the "patent cliff" (an estimated loss of 150 billion USD per year in turnover of big pharma companies by patent expiration within 2-3 years) attenuated.

In the process of drug development several checkpoints can be used to evaluate the probable translational success of a drug project. In 2009 a proposal for the scoring of the translatability of an early drug project was presented [2]. The score assesses the availability and quality of *in vitro *and *in vivo *results, clinical data, biomarkers, and personalized medicine aspects. The weights given to these different aspects reflect the particular importance in the translational process. The scores for the individual items are chosen between 1 and 5 and multiplied by the weight factors (/100). Any sum score above 4 is indicative of fair to good translatability and low risk. The predictive value of biomarkers is assessed using a special biomarker score which is described in ref. [3]. The biomarker score is included in the translatability score and comprises the evaluation of biomarkers in animal and human data, their proximity to the disease, accessibility and test validity parameters such as sensitivity and specificity. The use of an additional score for biomarkers besides the overall translatability score allows a more detailed analysis of the different facets of biomarker development, including *in vitro *data, animal models, human data, reproducibility etc. especially concerning the particular biomarker while the overall translatability score analyses the whole developmental process and includes further aspects as model compounds and personalized medicine. The importance of biomarkers in drug development is reflected by the high weight of this point in the overall translatability score, and the related biomarker scoring process is an important part thereof. Thus, this single most important partial aspect of translatability scoring deserves a structured approach by itself.

In this work, the two scores were applied to eight drugs either already approved for the market or failed during the developmental process. Published data were retrieved by entering the name of the drug into Medline, Biosis and Current Contents. All hits were screened for data applicable to the biomarker and translatability scores, and literature used for the assessment cited in the elaborate Tables 1 and 2. Each drug was fictively assessed after completion of phase II trials (publication date of references used or first public announcement of study results prior to the start of phase III as far as known) to answer the question: at which risk would this compound be taken to phase III? As an exception, gefitinib, a drug now approved for the treatment of lung cancer, was also analysed after the demonstration that an activating EGFR mutation is important for the clinical response which was published shortly after phase III studies had begun.

**Table 1:** Biomarker scoring for six drugs according to [3]

Points to evaluate	Dabigatran	Ipilimumab	Gefitinib	Gefitinib*	Vilazodone	Latrepirdine	Semagacestat
1	5 [4]	3 [5]	5 [6,7]	5 [6-10]	3 [11]	-	5 [12,13]

2	5 [4]	1 [5]	3 [6,7,14]	4 [6,7,10,14]	1 [15]	-	5 [12,13]

3	3 [16,17]	5 [5]	3 [1]	5 [1]	1	-	3 [18,19]

4	3 [20,21]	2 [5]	5 [1]	5 [1,10,22]	1	-	0 [23]

5	1	5 [5]	5 [24,25]	5 [8,9]	5 [26,27]	-	1 [23]

6	10 [20,21]	4 [5]	10 [24,25]	10 [8-10,22,28]	6 [26,27]	-	0 [23]

7	4 [29]	5 [5]	4 [30]	5 [28,30]	1	-	1 [23]

8	3 [31]	4	4	5	1 [32]	-	5 [33,34]

9	3 [35]	4	4	5	1 [32]	-	4

10	5 [36]	5 [1,5]	5 [1]	5 [1]	5 [11]	-	4

**Sum**	**42**	**38**	**48**	**54**	**25**	**0**	**0**

*after the development of the pivotal biomarker (EGFR mutation status)

The leading biomarkers evaluated were: aPTT for dabigatran, immune related response criteria for ipilimumab, effects on tumor growth for gefitinib, tumor growth and mutation status of EGFR for gefitinib, Hamilton Rating scale for depression-17 for vilazodone, none for latrepirdine, and effects on amyloid plaques for semagacestat.

Points to evaluate:

1. Are animal or in vitro data available?

2. How many species have been tested positively?

3. Are the animal models enough to reflect human disease?

4. Is there corresponding clinical data?

5. Are human data available?

6. Human data classification (2x)

7. Does the biomarker represent a pivotal disease constituent?

8. What is the statistical predictability?

9. What is the accuracy or reproducibility of the assay?

10. How accessible is the specimen?

**Table 2:** Assessment of translatability for eight drugs according to [2].

Compound	Dabigatran	Ipilimumab	Gefitinib	Gefitinib*	Vilazodone	Latrepirdine	Semegacestat	**Torcetrapib **[37]	**Varenicline **[37]
***Aspect***									

**Starting evidence**									

*In vitro *data Including animal genetics	0.1 [4]	0.06 [38]	0.1 [6,7,39,40]	0.1 [6,7,39,40]	0.08 [41]	0.06 [42-44]	0.1 [12]	0.1	0.1

*In vivo *data Including animal genetics	0.15 [4]	0.06 [45,46]	0.15 [6,7,14]	0.15 [6,7,14]	0.12 [41,47,48]	0.06 [42,49]	0.15 [12,50]	0.15	0.15

Animal disease models	0.09 [51]	0.06 [1,52]	0.09 [1]	0.09 [1]	0.06 [41,47,48,53]	0.06 [49]	0.03 [18]	0.12	0.15

Data from multiple species	0.15 [4]	0.09 [46]	0.06 [54]	0.06 [54]	0.06 [41,55]	0.03 [42]	0.15 [12]	0.03	0.15

**Human evidence**									

Genetics	0.05	0.05	0.05	0.25 [8-10,22,28]	0.05	0.05	0.25 [56-59]	0.05	0.05

Model Compounds	0.52 [60]	0.52 [61]	0.13 [54]	0.13 [54]	0.65 [62-64]	0.13 [42,43,65,66]	0.13		0.65

Clinical trials	0.52 [31,67]	0.65 [68-74]	0.26 [24,25,75-78]	0.65 [8,9,24,25,75-81]	0.26 [15,82]	0.13 [15,42,83]	0.39 [50,84,85]	0.26	0.52

**Biomarkers for efficacy and safety prediction**									

Biomarker Grading	0.96 [35,36]	0.96 [5]	1.2 [24,25]	1.2 [8,9,24,25]	0.72 [26,27]	0	0 [23]	0.48	1.2

Biomarker development	0.26	0.52 [5]	0.13 [24,25]	0.65 [8-10,22,28]	0.13 [26,27]	0	0 [23]	0.26	0.52

**Proof-of-mechanism, proof-of-principle and proof of concept testing**									

Biomarker strategy	0.2 [35,36]	0.2 [5]	0.05 [24,25]	0.25 [8-10,22,28]	0.15 [26,27]	0	0 [23]	0.1	0.25

Surrogate or endpoint strategy	0.4	0.4 [5]	0.24 [24,25]	0.32 [8-10,22,28]	0.24 [26,27]	0	0 [23]	0.16	0.32

**Personalized medicine aspects **									

Disease sub-classification and responder concentration	0.12	0.03	0.03	0.15 [8-10,22,28]	0.03	0.03	0.03	0.09	0.03

Pharmacogenetics	0.25	0.05	0.05	0.25 [8-10,22,28]	0.05	0.05	0.05	0.15	0.05

**Sum**	**3.77**	**3.65**	**2.54**	**4.25**	**2.6**	**0.60 (0)**	**1.28 (0)**	**1.95**	**4.14**

Data for torcetrapib and varenicline are taken from [37].

*after the development of the pivotal biomarker (EGFR mutation status)

Though chosen for this relatively late stage in development, the scores should be applied much earlier in preclinical and help to prioritize at this early stage already [2]. However, for better comparability we fictively chose the phase II/III transition; case studies at much earlier stages would have to be done separately.

It is obvious that not all relevant data are present in the public domain; this limitation of the present study has to be acknowledged.

Drugs from different therapeutic areas (anticoagulation, cancer, CNS-related diseases, and cardiovascular disease) have been evaluated. It is obvious that oncology projects, due to valuable biomarkers, show a much lower translational risk than candidates in the CNS field in which reliable, powerful biomarkers (and animal models) are largely missing.

## Case studies

### Dabigatran

Dabigatran (marketed as Pradaxa^® ^by Boehringer Ingelheim) was approved in the EU for the prevention of deep vein thrombosis after hip or knee operations in 2008. It is an orally applicable direct thrombin inhibitor [31,42]. In the present case study the development of dabigatran for the prevention of strokes in patients with atrial fibrillation (AF) was analysed. The unmet clinical need for stroke prevention in patients with AF is high, as the established long-term treatment by vitamin-K-antagonists (VKA, warfarin, phenprocoumon) is relatively unsafe and difficult to handle. VKA require continuous monitoring of the coagulation status of the patient, while dabigatran can be used without routine monitoring at a fixed dose supporting patient compliance. Another therapeutic option is acetylsalicylic acid, which is not very effective [86]. Direct thrombin inhibition in anticoagulation has already been validated and is the target of the parenteral anticoagulants hirudin and bivalirudin [60].

No good animal model of atrial fibrillation exists in general [16,51], but as dabigatran has been proven to prevent venous thromboembolism, several biomarkers for the monitoring of coagulation could be used for its development in the new indication. Activated partial thromboplastin time (aPTT) has been used as a biomarker of dabigatran effects in several species [4] and clinical trials [31,67]. Consequently, aPTT was assessed in the biomarker scoring here (Table 1). The correlation of aPTT with thrombosis and bleeding in a population with AF had not been clearly established at the time of the studies; this is reflected in the biomarker score, especially in items 4 and 5 (low scores of 3 and 1). Nevertheless, anticoagulation is successful in preventing stroke in AF [29] and aPTT received a total score of 42 indicating a biomarker of high value for translational risk prediction (Table 1).

The PETRO study was the first phase II trial which was conducted to analyse the effect of dabigatran in AF [67]. In the PETRO-EX study the optimal therapeutic dose of dabigatran was found to be 150 mg twice daily or 300 mg once daily. The phase III RELY study [87] verified the results of the phase II trials and was the basis for the decision of the FDA to approve dabigatran for the stroke prevention in patients with AF in September 2010.

The development of dabigatran for AF was done at relatively low risk as several features of dabigatran like safety and the effect on coagulation had already been investigated in earlier studies [31,88]. This is reflected by high translatability scores for the items model compounds, clinical trials, biomarker grading and surrogates in Table 2. This case study represents the development of a new therapeutic indication of an already approved drug, which is of much lower risk than the development of a new drug for a new application. This lower risk is clearly indicated by the overall translatability score, which is 3.77 (Table 2) and therefore indicates mean to fair translatability.

### Ipilimumab

Ipilimumab, marketed as Yervoy^® ^by Bristol-Myers Squibb, is the first therapeutic agent which increases survival time in patients with metastatic malignant melanoma, the leading cause of death from skin disease [89]. Previous phase III studies failed to show a survival benefit [90-92]. Two therapeutic compounds already approved by the FDA for the treatment of stage IV melanoma, an old chemotherapeutic drug (dacarbazine) and high dose therapy with the immune stimulant interleukin-2 failed as well [93]. Additionally, high dose interleukin-2 therapy has many adverse effects, so that excellent cardiovascular and pulmonary functions are required for its safe use [68,94,95]. Therefore, the unmet clinical need is high for the treatment of metastatic malignant melanoma.

Ipilimumab is a fully human monoclonal antibody (IgG) blocking CTLA-4 (Cytotoxic T-Lymphocyte antigen 4) to promote antitumor immunity. It acts as a negative regulator of T-cell activation [38]. *In vivo *studies showed that blocking CTLA-4/B7 interactions in murine models induced rejection of different transplantable tumors, like colon cancer, prostate cancer, lymphoma and renal cancer [96]. *In vivo *administration of anti-CTLA-4 antibodies to mice results in rejection of tumors, including pre-established tumors. Further, immunity against a secondary exposure to the tumor was detected [45]. Engagement of CTLA-4 on the surface of activated T-cells by co-stimulatory molecules inhibits IL-2 and IFNγ production upon T-cell receptor engagement. Blockade of this negative signalling with CTLA antibodies may result in further activation of activated T-cells and consequently lead to antitumor activity [93]. Phase I and II trials showed that ipilimumab is effective in patients with melanoma [97,68-74].

In a phase II trial, "immune related response criteria" (irRC) for the evaluation of immune-based cancer therapies were studied [5]. These criteria were newly defined in a series of workshops on immunotherapeutic agents in cancer patients. This was inevitable as the criteria normally used for the evaluation of anticancer therapeutics, the WHO criteria and RECIST (Response evaluation criteria in solid tumors), are not suitable for the evaluation of immune-based therapies [5]. The clinical effect of ipilimumab not acting on the tumor itself is delayed and tumor growth may continue during the first weeks of treatment. Therefore, the patients seem to show progressive disease which would be typically defined as drug failure by the WHO criteria and RECIST. The newly defined criteria include total tumor burden, which is calculated by summation of the product of the perpendicular diameters of measurable index lesions, time point assessments, and overall response. Further, new lesions are taken into account [5]. Evaluation of the irRC using the biomarker score results in the classification as a medium-high-value marker (score: 38) (Table 1).

Immune-response related adverse events frequently occur in patients treated with ipilimumab, which were found in all trials [97,68-74]. Diarrhea and colitis as gastrointestinal adverse effects, hypophysitis as endocrine dysfunction, ocular toxicities, and pancreatitis are the main adverse effects. Despite the high risk of adverse effects the drug was approved by the FDA in March 2011. In the overall translatability scoring ipilimumab reaches a score of 3.65, which indicates a mean to fair translatability (Table 2). The high scores for the newly developed biomarker (reflected in biomarker grading and development), for the surrogates, the promising results in the clinical trials and the high score for model compounds are the main contributors to this relatively high score.

### Gefitinib

Gefitinib (Iressa^®^) was approved for treatment of non small lung cell cancer after failure of docetaxel- or platinum-based chemotherapy [98] by the FDA in 2003 under the auspices of the accelerated approval program. This program gives patients with serious or life-threatening diseases earlier access to promising new drugs. Gefitinib is a selective reversible inhibitor of the EGFR (epidermal growth factor receptor) tyrosine kinase domain and inhibits the anti-apoptotic RAS signal transduction cascade [99]. The drug leads to an increased survival time in some patients with non small cell lung cancer [8,9]. The unmet clinical need was high as patients diagnosed with lung cancer expose a bad prognosis: 5- year survival rate is just 16%.

Several studies showed that the drug only works in patients with activating mutations in the EGFR [10,22,28]. 10-15% of the patients in Western countries show these mutations. 71% of the patients carrying the mutation respond to treatment, but only 1% of the patients without this mutation. The responsible mutations include deletions in exon 19 (46%), duplication and insertion in exon 20 (9%) or point mutations in exon 21 (39%). Analysis of gene copy numbers of the EGFR has not consistently shown that this mutation is also involved in a larger response to gefitinib in the treatment of non small lung cell cancer after failure of docetaxel--or platinum-based chemotherapy [10,22,98]. The dependence of the efficacy of gefitinib on the mutation status was detected by *in vivo *and *in vitro *studies [10] after the start of phase III trials. Consequently, the failure of the first phase III trials (INTACT I [79] and II [80]) was due to a comparatively low rate of patients with EGFR mutations. In the ISEL trial [81] also no increase of overall survival time was detected, but a substudy revealed that neversmokers had an increased survival time (8.5 vs 5.5 months). This effect was even greater in patients of Asian origin (9.5 vs 5.5 months). Asian populations have much higher rates of EGFR activating mutations. In response, the FDA revoked the accelerated approval of 2003 in 2005, and limited the indication to patients who were already on the drug and had benefited from it. In the INTEREST trial [8] the impact of activating EGFR mutations was clinically shown by a significantly higher response rate (42.1 vs 21.1%). Accordingly, the IPASS study [9] on Asian patients demonstrated a higher response rate for gefitinib versus standard therapy and patients without the mutation did not respond to gefitinib. The EMEA approved gefitinib for the treatment of non small cell lung cancer for patients carrying an activating EGFR mutation in 2009.

In the evaluation of these studies the inclusion of the EGFR mutation status into the biomarker panel improved the overall translatability score from 2.54 to 4.25 (Table 2). This increase reflects higher individual scores for biomarker grading, biomarker development, strategy, clinical trials and personalized medicine items. The biomarker score alone would predict a high translatability also for the use of tumor growth as this is a widely used biomarker. Only the translatability score considers the importance of the mutation. This case clearly shows that the use of both scoring systems is important to more accurately predict success of the particular project. As already mentioned, gefitinib is an example of a drug in which personalized medicine aspects play a pivotal role for the responder rates. Instead of being a blockbuster with an indication for all lung cancer patients, gefitinib is only effective in 10-15% of the patients in Western countries. The company decided to push the compound before personalized medicine issues had been solved which were likely to exist. Therefore, the blockbuster-type approach was doomed to fail. The case of gefitinib is a good example for the trend to use more genetic biomarkers to aid personalization instead of the development of block buster drugs in the field of oncology (and elsewhere). Additionally, the development of companion diagnostics is an important field of drug development, underlining the importance of biomarkers again, especially in oncology. Therefore the item for personalized medicine in this field is of great importance and may eventually be weighted higher in oncology.

### Vilazodone

Vilazodone (5-(4-[4-(5-cyano-3-indolyl)-butyl]-1-piperazinyl)-benzofuran-2carboxamide hydrochloride) was approved by the FDA in January 2011 for the treatment of major depressive disorder and is marketed as Viibryd^® ^by Clinical Data Inc. Despite the availability of approved drugs for the treatment of major depressive disorder, many patients do not adequately respond to these therapies and therefore new, more effective drugs are needed. Recent experiments have shown that the administration of 5HT_1A _(5-hydroxytryptamine) antagonists augments the effects of SSRIs on extracellular 5-HT [100,101]. Vilazodone is a selective serotonin reuptake inhibitor (SSRI) (IC_50 _= 0.2 nM) and a 5-HT_1A _receptor partial agonist (IC_50 _= 0.5 nM; IA = ~60-70%) [41,55].

For a number of reasons, the development of drugs acting at the CNS carries a greater translational risk than, for instance, the development of drugs against malignancies. These reasons include that

i) the animal models used to analyse the effect of drugs in psychiatric disease (shock probe test, predator stress model, forced swimming test, ultrasonic vocalisation model in rodents) are not very effective to predict drug efficacy in patients,

ii) biomarkers used in patients with major depressive disorder (Hamilton rating scale for depression, Montgomery-Asberg Depression rating scale, Clinical Global Impressions Improvement, Hamilton Anxiety Rate Scales) are weak and can only be used in patients, but not in animals.

Thus, these biomarkers are not potent to predict risk at early stages of drug development. Further, their statistical predictability and reproducibility are not high [32]. In consequence, the translational score for CNS drugs is low in most cases and such projects remain at high risk.

Vilazodone was evaluated in five phase II randomized, placebo-controlled studies in patients with major depressive disorder by Merck and GlaxoSmithKline. Three of the trials used active comparators and all employed the Hamilton Rating Scale for Depression-17 (HAM-D17) [11] as primary outcome [15]. Therefore this scale was also used in the biomarker scoring in this work (score: 25, Table 1). The three studies that had an active control failed to show superiority and the remaining two studies were negative (http://www.accessdata.fda.gov/drugsatfda_docs/nda/2011/022567Orig1s00CrossR.pdf). Despite these negative results Clinical Data Inc. obtained an exclusive license and showed statistically significant efficacy against placebo in a phase III trial [102]. Further biomarkers to predict therapeutic efficacy were developed (key genes in the 5-HT pathway) (reviewed in [103]). However, the biomarkers failed to show an association with the response to vilazodone in a second phase III trial. The biomarker have not been published yet; they are not used to identify responders as it has been shown that vilazodone is beneficial to a broader group of patients than defined by the use of these biomarkers [103].

Despite the failure of the phase II trials and the weak biomarker profile of this project the drug was finally approved after the two successful phase III studies. Vilazodone belongs to the widely used class of SSRIs and therefore shows the characteristics of the development of a "me too" compound, which is reflected by the high score for model compounds in the overall translatability score. The new feature of a 5-HT_1A _receptor partial agonist increases the translatability of this project since it was already shown that combining pindolol, a mixed 5HT1A/ß-adrenergic receptor partial agonist, with SSRIs enhances the increase of extracellular levels of 5-HT in preclinical studies [62] and produces a more rapid onset of antidepressive effects [63,64]. Further, compared to other SSRIs on the market, vilazodone shows less impairment of sexual function [15]. Despite the characteristics of a me-too compound, the score for overall translatability of vilazodone indicates a poor to intermediate translatability and, thus, comparably high risk (score: 2.6, Table 2) due to the lack of powerful biomarkers in this field. This example shows that even me-too compounds may have a high translatability risk if powerful biomarkers are missing. Despite this low score, the compound was finally approved against all odds and shows that even low score projects may eventually be successful. The item on model compounds in the translatability score was ranked high; this fact may be taken as a starting point for the improvement of the scoring tool in that the weighing of model compounds should be even higher than in the present algorithm.

### Latrepirdine

Latrepirdine (2,3,4,5-tetrahydro-2,8-dimethyl-5-[2-(6-methyl3-pyridinyl)ethyl]-1H-pyrido[4,3-b] indole, provided as the dihydrochloride salt) was formerly used as antihistaminic drug and marketed as Dimebon^® ^[42], but was removed from the market due to the development of more selective drugs in the field. Recently it was evaluated as a drug against Alzheimer's disease (AD). The unmet clinical need is high for new drugs against AD as no efficient causal treatment exists so far [104]. The major problem in the development of drugs against AD seems to be the lack of knowledge about the exact pathogenetic mechanisms resulting in AD.

Latrepirdine was shown to act as a cholinesterase and NMDA inhibitor, the two mechanisms of action of existing symptomatic AD drugs [42,43,65]. Newer studies have demonstrated that its primary action in AD relates to the stabilization of mitochondrial function. Evidence from *in vitro *studies suggests that latrepirdine might protect against amyloid-β(Aß)-mediated toxicity in primary neuron cultures and improve mitochondrial function in cultured cells [42-44]. However, it is unclear whether latrepirdine can exert a disease-modifying activity *in vivo *and improve AD neuropathology and/or clinical symptoms in animal models of AD. In contrast to many other AD drugs in the pipeline, latrepirdine's action is not based on the reduction of amyloid plaques. The importance of plaque formation in AD is controversially discussed, and the opinion that the overproduction and accumulation of Aβ in the brain are key pathogenic events in AD progression is increasingly questioned [105]. Concerning the disease modifying activity of latrepirdine no data are available from the different clinical trials as only the 11-item ADAS-cog was used as primary outcome. The 11-item ADAS-cog is only available at the human level and risk assessment at early stages is hampered by the lack of appropriate animal biomarkers and animal models. In the clinical trials no biomarker for the disease modifying action has been used. Therefore, biomarker grading and strategy represent knock-out criteria in the overall translatability scoring (Tables 1 and 2) supporting a no-go decision at an early stage of development. This example supports the view that biomarkers are the single most important parameter for go/no-go decision at the transition from preclinical to clinical and early clinical to late phase where the weight is more on clinical and safety biomarkers and the overall strategy for their use.

As latrepirdine had been available as antihistaminic drug several years ago, its safety profile seemed to be established. Phase I [42] and II [83] trials were encouraging as latrepirdine improved the clinical course of the patients. Surprisingly, in the subsequent phase III trial the drug failed to show a significant effect compared to placebo [106].

The failure of this drug was likely (translatability score 0.6, or 0 if knock-out is accepted) as the pathogenetic mechanisms are not understood for AD and no powerful biomarkers exist. Using the scores during the developmental process of the drug might have prevented the expenditures for its late clinical development. The primary development of a biomarker to assess the disease progression and its therapeutic modification would be important, but requires knowledge about AD pathogenesis and the way of action of latrepirdine in AD.

### Semagacestat

Semagacestat is a gamma-secretase inhibitor and inhibits the final step in Aß-protein synthesis as putative target for AD treatment. Therefore, unlike latrepirdine the mode of action is known for semagacestat. The molecule rapidly reduces Aβ concentrations in the brain, cerebrospinal fluid (CSF), and plasma of transgenic V717F human amyloid precursor protein mice (PDAPP mice) [12] and in the plasma of humans [13]. In the development of semagacestat amyloid plaques have been used as biomarker like in many other AD studies. Until now, all other studies based on this biomarker failed supporting the assumption that measuring the formation of plaques is insufficient to predict therapeutic success. The importance of plaque formation in AD is controversially discussed, and the opinion that the overproduction and accumulation of Aβ in the brain are key pathogenic events in AD progression is increasingly questioned [105]. Accordingly, plaques can be reduced by semagacestat, but the symptoms are not improved in treated AD patients. The plaques might be useful to identify patients with AD [107] (disease indicator), but they are no valuable tool to predict treatment success (disease factors). Additionally, imaging methods to detect the plaques are quite expensive [108]. Therefore the scoring for plaques as biomarkers contains knock-out features for corresponding clinical data and human data classification and is rated at 0 in the overall translatability score (Tables 1 and 2). Additionally, skin cancer was observed as a severe adverse effect of the treatment with semagacestat; cognitive function even worsened in the phase III trial.

Semagestat is another example of a failed drug development in the AD field, with a high risk translatability score of 1.28 (or 0 if the knock-out criterion is accepted).

The major implication of the assessment of these two potential AD drugs is the fact that the etiology of AD is not yet understood and Aß targeted therapies are likely to attack an epiphenomenon. This gap of knowledge is mainly reflected by the lack of a biomarker placed more proximal in AD etiology. Further studies are needed to analyse the pathogenesis of the disease and to develop suitable biomarkers.

The Coalition Against Major Diseases (CAMD) has released a database on 4,000AD patients who have participated in 11 industry-sponsored failed clinical trials. Publishing of negative trial results is important to prevent further trials from failing and is an important achievement in the development of drugs against AD.

Two further drugs (torcetrapib and varenicline) have been scored for translatability in a recent publication [37]; the related scoring items are cited here for comparison and the widening of the spectrum of therapeutic areas.

### Torcetrapib

Torcetrapib was developed to treat hypercholesteria and prevent cardiovascular disease. It inhibits cholesteryl ester transfer protein (CETP) resulting in increased concentrations of HDL-cholesterol ('good cholesterol'). CETP inhibitors increased HDL-levels in various animal models and early human trials, and could even prevent diet-induced atherosclerosis in NZW rabbits, albeit not in other animal models. The development of torcetapib was based on the hypothesis that an intervention leading to increased levels of HDL-cholesterol should be beneficial for the patients. It is now known that the use of HDL as a biomarker was too positive and other biomarkers like intima-media thickness should have been used to predict efficacy to prevent cardiovascular disease. The test-run of the scoring proposal at a fictive knowledge status prior to the ILLUSTRATE results produces a sum score of 1.95 (Table 2), clearly indicating a high-risk translational project [37].

### Varenicline

Varenicline is a novel drug to aid smoking cessation and was developed by Pfizer. The drug partially agonizes the nicotinic receptor and, thus, reduces craving for smoking. As receptors are occupied, a new cigarette respectively the nicotine contained is ineffective ("antagonized").

The translational evidence was based on isolated receptor subtypes cloned from animals and humans and on valid animal models for nicotine dependence and nicotine side effects (e.g. hypothermia). These are absent in limited ceiling effects of partial agonists. Biomarkers at the animal level (behavioural measures) and comparably simple clinical studies on smoking habits and psychometric scales for craving and satisfaction had been established and validated at the time of varenicline translation. This included the use of model compounds (especially bupropion). The fictive translatability score prior to the pivotal Phase III trials was 4.14, indicating a high likelihood of translational success [37].

## Conclusions and outlook

The overall translatability score awaits validation. The present work demonstrates that the scoring process seemingly produces plausible results retrospectively which is encouraging. The scoring system clearly shows that the early development and use of powerful biomarkers considerably decreases the risk in drug development. In general, stronger biomarkers exist to develop drugs against malignant than CNS diseases.

All limitations of retrospective analyses apply to the case studies, and biases in the scoring of the individual items reflecting the final drug approval status cannot be excluded. Careful referencing and, thus, transparency of decisions are important in this process. The results and their open traceability together with plausible results should encourage industry and--perhaps--even public funding agencies to plan and finance a prospective validation study. Unfortunately, this study would probably take a decade to produce valuable results as drug development cycles require that much time. Nevertheless, the system already now appears as a valuable tool to calculate the risk of a current drug development project. Further, the scores provide guidance to identify weaknesses and opportunities in drug development and, thus, may lead to strategy adjustments, with the pre-emptive development of biomarkers as a prominent example.

The score carries all features of likelihood determinations, and a low translatability score does not preclude success (as for vilazodone), and a high score does not guarantee it either. It should not be seen as a static model, but needs to be developed and adapted to different therapeutic areas; for example, the item on personalized medicine should be weighed higher in oncology than cardiovascular medicine. As mentioned above, the weight for model compounds should be reconsidered and--depending on the therapeutic area--probably increased.

Therefore, as a future task scoring parameters and weights should be adjusted according to the compound class and the targeted system pathways. Due to the high diversity of drugs developments in different diseases, the score must be flexible and dynamic to be applicable to most situations. The validation and adaptation of the score might be best placed in a pre-competitive environment such as a consortium of researchers from big pharmaceutical companies.

Though the individual items and weight factors might be debatable, the major impact of the scoring process as such should be seen in the establishment of a structured profiling and transparent, vigorously scientific, objective reasoning as opposed to an irreproducible, subjective and unstructured "gut-feeling" decision taking. The metric values seem less important than the structuring potential of this approach.

As a second important aspect discussed in detail in [2] the scoring at the early phase identifies weaknesses of a given project at an early stage and, thereby, may strategically help to define the need of particular clinical studies for sufficient proof, may indicate the need for the development of companion diagnostics or rigorous patient stratification. These strategies would then aim at improving weak scores at the first instance, but ultimately more important, help to reduce risks. This will be part of the evaluation of costs, risks and ROI to make go/no-go decisions in a qualified way.

In conclusion, eight retrospective case studies of translatability scoring produced scores which are in line with success or failure as project outcome, underlining the plausibility and utility of the approach. The score should be developed, adapted and prospectively validated.

## Competing interests

AW has nothing to declare. MW was employed by AstraZeneca R&D, Mölndal, as director of discovery medicine (= translational medicine) from 2004-2006, while on sabbatical leave from his professorship at the University of Heidelberg. After return to this position in January 2007, he received lecturing and consulting fees from Sanofi-Aventis, Novartis, Takeda, Roche, Pfizer, Bristol-Myers, Daichii-Sankyo, Lilly and Novo-Nordisk.
